# Computer Vision Syndrome Among Saudi University Students: A Cross-Sectional Analysis of Risks and Discipline Variations

**DOI:** 10.3390/healthcare13212798

**Published:** 2025-11-04

**Authors:** Osama Albasheer, Mohammad A. Jareebi, Raghad M. Alnami, Asma M. Soweedi, Saja S. Alqahtani, Amal M. Ageeli, Fai Y. Arif, Aghadir H. Judayba, Alanood M. Hakami, Dhiyaa A. H. Otayf, Saja A. Almraysi, Ahmed Y. Najmi, Ahmad Y. Alqassim, Majed A. Ryani, Ahmed A. Bahri

**Affiliations:** 1 Family and Community Medicine Department, Faculty of Medicine, Jazan University, Jazan 45142, Saudi Arabia; oalbasheer@jazanu.edu.sa (O.A.); aalqassim@jazanu.edu.sa (A.Y.A.); majedryani@gmail.com (M.A.R.); dr.bahri2010@gmail.com (A.A.B.); 2 Faculty of Medicine, Jazan University, Jazan 45142, Saudi Arabia; raghad.m.alnami@gmail.com (R.M.A.); sewdiasma@gmail.com (A.M.S.); sajasaeedq@gmail.com (S.S.A.); amalageely0123@gmail.com (A.M.A.); faiaref89@gmail.com (F.Y.A.); agadirjudayba@gmail.com (A.H.J.); anoodanod2@gmail.com (A.M.H.); dhiyaaot@gmail.com (D.A.H.O.); sajaalasiri1@gmail.com (S.A.A.); ahmednajmiofficial@gmail.com (A.Y.N.)

**Keywords:** computer vision syndrome (CVS), electronic devices (EDs), Jazan University, prevalence, risk factors, university students

## Abstract

**Background and Objectives:** Computer Vision Syndrome (CVS) has become a major health problem among university students as a result of extensive electronic device use, but there is limited in-depth risk factor analysis by academic disciplines. The purpose of this study was to determine CVS prevalence, identify risk-associated factors, and investigate discipline-specific differences among university students. **Methods:** A cross-sectional study was conducted at Jazan University among 427 students of six academic disciplines between 2023 and 2024. Questionnaires validated by collecting demographics, electronic device usage patterns, eye care practices, and CVS symptoms were used to assess the data. Statistical analyses involved chi-square tests and multivariable logistic regression with significance at *p* < 0.05. **Results:** Prevalence of CVS was at epidemic proportions at 89.7% (95% CI: 86.8–92.6%), which was much higher than global averages. Considerable inter-disciplinary heterogeneity occurred, from 95.3% in Computer Science to 75.4% in Arts and Humanities students. A strong dose–response gradient was found for duration of device use: 3–4 h (OR = 4.13, 95% CI: 1.13–5.57), 5–6 h (OR = 5.31, 95% CI: 1.46–9.86), and ≥7 h per day (OR = 6.25, 95% CI: 1.74–8.01) versus 1–2 h use. Students >24 years old demonstrated a very high risk (OR = 9.73, 95% CI: 1.53–19.65). Headaches were the most common symptom (68.0%), and adoption of protective measures was low. **Conclusions:** This work demonstrates epidemic-level prevalence of CVS with unequivocal dose–response relationships and discipline-specific risk patterns, offering evidence-based targets for immediate campus-wide interventions and identifying a vital post-pandemic public health challenge meriting immediate attention.

## 1. Introduction

Electronic devices (EDs) are now an integral part of individuals’ lives, particularly for students. It provides significant support in day-to-day tasks and allows students to access a great amount of knowledge and information [1]. Studies reveal electronic devices (EDs) are now an indispensable component of children’s daily lives, with 2- to 4-year-olds spending an average of 2.5 h watching screens every day, well beyond the one-hour mark that has been recommended. The typical household has 10 electronic devices, of which 4 are usually available to children. The pervasive early exposure is highly suspected to have many visual health risks [2]. The children were less likely to play outdoors, and over 50% spent less than one hour per day outside [3]. This behavior shift creates perfect conditions for Computer Vision Syndrome (CVS) development: extended near work on digital screens triggers accommodative stress and reduced blinking, while limited outdoor exposure decreases the protective effects of natural light exposure on visual development. The exposure and improper use of these devices can lead to multiple health consequences. A prior work indicates that 61% kids and 78% teens who engaged for more than 2 h a day on EDs during school days and weekends/holidays were associated with increased frequency and severity of musculoskeletal, optical, and poorer psychosocial health for device use [4]. Another recent systematic review discovered that overuse of ED has the potential to contribute to maladaptive screen use behaviors among children, which have been associated with numerous negative consequences [5]. These include physical problems such as obesity, psychological problems like language delay, attention deficit hyperactivity disorder (ADHD), social health outcomes, and impaired executive functions [5]. Among all these diseases of health, one frequent complication of misuse and overuse of ED is computer vision syndrome (CVS), which is 66% frequent among children and teens [5].

CVS, as described by the American Optometric Association, is a group of eye- and vision-related problems resulting from prolonged use of computers, tablets, e-readers, and cell phones. This aligns closely with Rosenfield’s description of CVS as a constellation of ocular and visual symptoms associated with near work on digital displays [6]. It produces symptoms such as eye strain, dryness, headaches, blurred vision, and neck strain through constant focusing, decreased blinking, and bad posture [7]. In addition to visual discomfort, CVS can lead to long-term conditions like disturbed sleep patterns because of exposure to blue light, greater stress from chronic discomfort, and lower productivity levels [8,9]. With time, the stress on the visual system can worsen latent conditions such as myopia progression, thus contributing to the worsening of eye health [10]. One study found that 20% of students presented with more than one symptom of eye issues, 9% complained of eye pain, 8% complained of dry eyes, and 6% complained of watering eyes and red eyes due to CVS associated with prolonged use of EDs [6]. These signs are divided into four categories: internal ocular signs (e.g., eye strain and ache), external ocular signs (e.g., dryness and burning, irritation), optical signs (e.g., double vision and blurred vision), and musculoskeletal symptoms (e.g., shoulder, neck, and back pain). These problems are mainly linked to eye fatigue, strain, ergonomics, and posture while using ED displays continuously [11,12].

Epidemiologically, the overall global prevalence of CVS was estimated at 66–69% in a systematic review and meta-analysis by Anbesu et al. [13]. Within Saudi Arabia, it was found that the prevalence of CVS in the general Saudi population was 77.6%, and the most commonly described symptom was eye burning (71%) [14]. A Saudi study calculated the prevalence of CVS among university students to be 82.2% [15]. CVS levels were specifically related to the duration of ED use, female gender, advanced age, and the existence of an eye pathology. In addition, college students are exposed to higher rates of CVS than others. Wang et al. explained that CVS was detected in 77.1% of undergraduate and 69.1% of medical students [16].

Though CVS is common among the population of Saudi Arabia, particularly among college students, information on this condition among Jazan University students is limited. This study addresses this gap by assessing the prevalence and determinants of CVS among Jazan University undergraduate students. Guided by the research questions: (1) What is the prevalence of CVS among students? (2) Which risk factors are associated with CVS? and (3) Are there differences in CVS prevalence across academic disciplines? This study aimed to estimate CVS prevalence, identify primary risk factors, and investigate variations across academic disciplines. The findings will inform preventive measures, awareness campaigns, and policy recommendations to mitigate CVS-related discomfort and enhance eye health among students, while providing a foundation for future research into digital eye strain interventions and guidelines for healthier screen practices in academic settings.

## 2. Materials and Methods

### 2.1. Study Design and Participants

**Figure 1:**
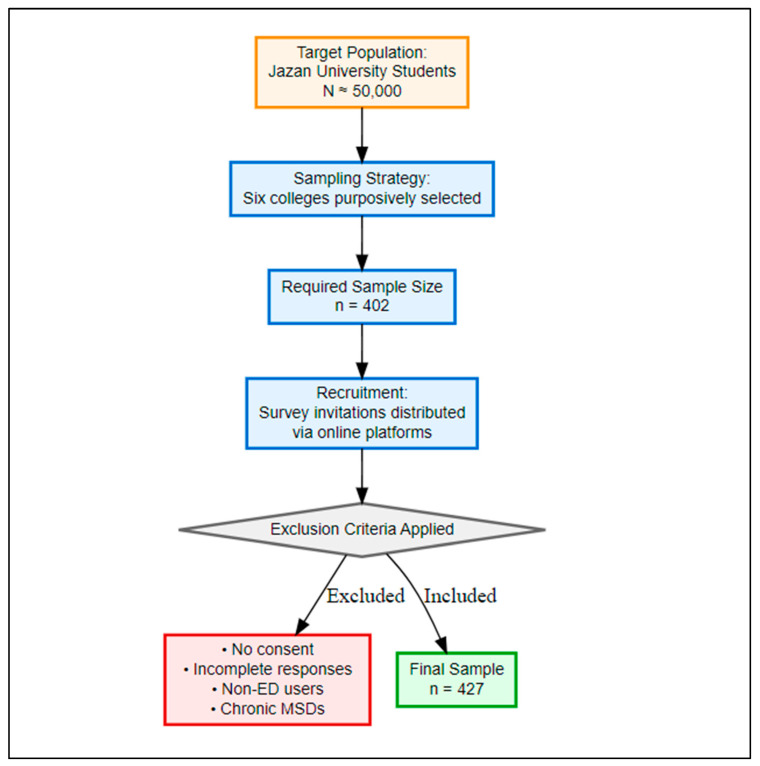
Flowchart of recruitment process, inclusion, and exclusion of study participants.

This is a cross-sectional, questionnaire survey (Supplementary File S1) that was carried out at Jazan University in the Jazan area of Saudi Arabia between August 2023 and June 2024. Jazan University comprises 23 colleges, and the target group was estimated to be approximately 50,000 students. For feasibility, six representative colleges (Computer Sciences and IT, Business Administration, Medicine, Applied Medical Sciences, Arts and Humanities, and Engineering) were purposively selected. These were chosen to reflect diverse academic demands and digital-device exposures, ranging from highly technology- and health-oriented fields to less device-intensive disciplines. Other colleges were not included due to feasibility constraints. This approach enabled meaningful cross-disciplinary comparisons, though it does not represent all university fields (Figure 1).

The minimum sample size was calculated using a conservative assumed proportion (*p* = 0.50), 95% confidence level (Z = 1.96), and a precision (margin of error, E) ranging between 5% and 8%, as commonly accepted for cross-sectional studies. The required sample sizes for these margins ranged from n = 384 (E = 5%) to n = 150 (E = 8%). Allowing an additional 5% for potential non-response, the minimum required sample was approximately 402 students. To enhance precision, the final achieved sample size was 427 students, exceeding the target.

The corresponding margin of error for the sample achieved and observed CVS prevalence (89.7%) was ±2.9% at 95% confidence, indicating greater precision than initially designed. The sample size calculation was cross-checked using the standard formula:

$$n=\frac{Z^{2}\cdot p\cdot \left(1-p\right)}{E^{2}}$$

where Z is the critical value corresponding to the selected confidence level (1.96 for 95% CI), *p* is the expected proportion (0.5), and *E* is the acceptable margin of error (5–8%).

### 2.2. Data Collection Tool

This research employed a validated, self-administered questionnaire that was distributed conveniently to the participants. The questionnaire had four parts: sociodemographic details, ED utilization-related variables, participants’ eye care behaviors, and CVS-related queries.

The sociodemographic data section consisted of queries about participants’ age, sex, college, and Grade Point Average (GPA).

The second part explored ED usage variables. It presented questions regarding the duration and state of ED utilization, distance between screen and facial distance, position of the device in relation to the face (above, below, or eye level), utilization of anti-glare screens and blue light glasses, adherence to rest breakdowns (after applying 20-20-20 rule), use in areas with plenty of light, and highest screen brightness settings. The 20-20-20 rule states that after 20 min of screen use, one should look at an object at least 20 feet away for 20 s. Participants self-reported their adherence to the 20-20-20 rule and the use of protective measures (e.g., anti-glare screens, blue-light filters) via questionnaire.

### 2.3. Measures

The third part questioned participants’ eye care habits. Participants were queried regarding headache experiences during ED use, the wearing of artificial eye drops (whether prescribed or not), and the wearing of glasses or contact lenses, as well as the rationale for wearing them.

The fourth part consisted of questions measuring the occurrence of CVS among study participants. The questionnaire was adapted from previously validated CVS instruments [17]. CVS prevalence was assessed using a symptom-based approach via questionnaire, consistent with the American Optometric Association definition. The complete questionnaire is provided as Supplementary Material (File S1). Nine CVS-related symptoms were assessed (teary eyes, dryness, irritation, retro-bulbar pain, eye strain, redness, blurred vision, hot sensation in the eyes, and headache). Each symptom was rated on a 0–5 Likert scale (0 = none to 5 = very severe). Participants reporting at least one symptom (score ≥ 1) during or after digital-device use were classified as having CVS. This operational definition is aligned with the American Optometric Association’s description and Rosenfield’s conceptualization of CVS.

To evaluate the clarity of the questionnaire, a pilot test was conducted with 30 participants. Two weeks later, the same group completed the survey again to assess test–retest reliability. The results demonstrated excellent agreement, with an intraclass correlation coefficient (ICC) of 0.86. Following the content validity assessment, the questionnaire was given to the target population, leaving out the pilot subjects. The online surveying method was employed to administer the questionnaire, and conveniently accessible through various social media platforms, such as WhatsApp, Telegram, X (Twitter), and Facebook, were utilized to communicate. Participants were informed of the purpose and objectives of the study prior to their consent to take part. In addition, every precaution was exercised to observe privacy and confidentiality throughout the study.

### 2.4. Statistical Analysis

Following data collection, the raw dataset was exported to Excel for validation, where it was examined for errors, inconsistencies, and missing values. Statistical analyses, including descriptive statistics, chi-square tests, and multiple logistic regression, were conducted using R software (version 4.2.3, R Foundation for Statistical Computing, Vienna, Austria). For multivariable logistic regression, variables with *p* < 0.20 in univariate analyses were included, with age and sex added a priori as confounders. The final model comprised demographics (age, sex, academic field), device-use duration, ergonomic practices, and protective behaviors (e.g., anti-glare screens, 20-20-20 rule). Adjusted analyses were performed, and the reported odds ratios (OR) represent associations after controlling for these specified confounders. Multicollinearity was checked (VIF < 5), missing data were handled by complete-case analysis, and model fit was assessed using R^2^. Descriptive statistics involved computing means, percentages, and standard deviations (SD), depending on the nature of each variable. Chi-square tests and multiple logistic regression are used to assess the associations among CSV and various influencing factors. A significant threshold of *p*-value < 0.05 was used for all statistical tests.

### 2.5. Ethical Approval

Ethical approval of this study was obtained from the Standing Committee for Scientific Research of Jazan University (Reference No. REC-45/05/856, dated 4 December 2023). Prior to consent being sought, participants were given a clear description of the purpose of the study, possible outcomes, and their rights, such as voluntary withdrawal, confidentiality, and protection of privacy. Personal identifiers were not gathered, and access to data was limited to the research team to ensure discretion. In addition, this cross-sectional study complied with the ethical standards of the Declaration of Helsinki and STROBE (Strengthening the Reporting of Observational Studies in Epidemiology) to provide clarity and completeness in reporting.

## 3. Results

### 3.1. Demographic Characteristics

This was a cross-sectional study involving 427 university students with equal gender distribution (50% male, 50% female). Most were 21–23 years old (59%), then 18–20 years old (35%), and ≥24 years old (6%). The students came from six colleges: Computer Sciences IT (20%), Business Administration (19%), Medicine (18%), Applied Medical Sciences (15%), Arts and Humanities (14%), and Engineering (14%). The majority of participants (69%) had high academic performance with a GPA ≥ 4.0 (Table 1).

### 3.2. Computer Vision Syndrome Prevalence by Academic Discipline

**Figure 2:**
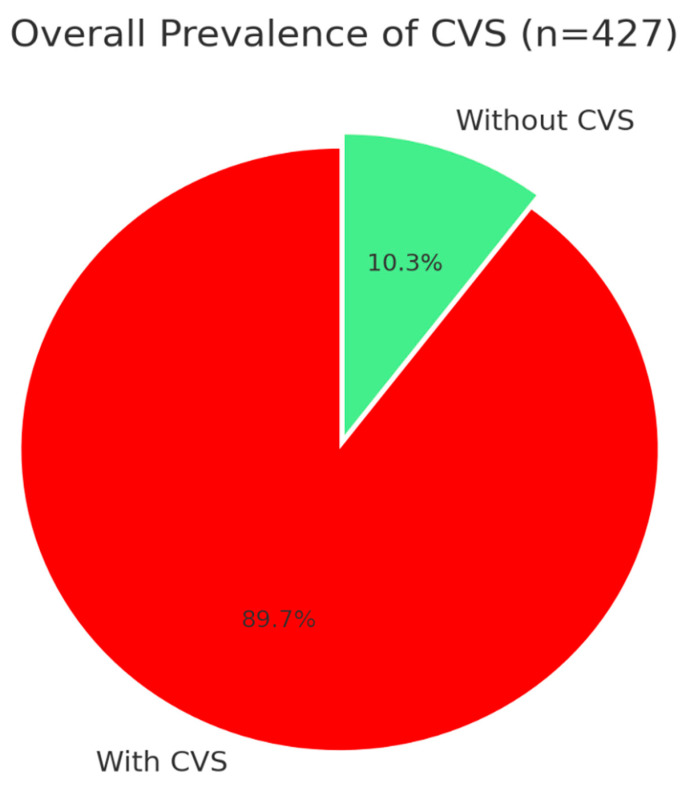
Prevalence of Computer Vision Syndrome (CVS).

The total prevalence of CVS was 89.7% (95% CI: 86.8–92.6%, n = 383/427) (Figure 2). Prevalence was determined using the operational definition described in Methods (participants reporting ≥ 1 symptom with score ≥ 1). High inter-disciplinary variation was found (χ^2^ = 18.42, *p* < 0.001), with technology and health sciences disciplines reporting uniformly higher prevalence rates than humanities (Table 2).

### 3.3. Electronic Device Usage Patterns and Dose–Response Relationship

A strong dose–response association was demonstrated between the number of hours daily device use and CVS prevalence. Models were adjusted for age, sex, academic field, device-use duration, ergonomic practices, and protective behaviors (Table 3). The dose–response examination showed that in comparison to students spending 1–2 h daily on devices, the risk was very high: 3–4 h demonstrated 4.05-fold higher odds, 5–6 h demonstrated 5.68-fold higher odds, and ≥7 h had 7.15-fold higher odds of CVS development.

### 3.4. Device Usage Practices and Protective Behaviors

Practices of device usage were analyzed and found to be common suboptimal ergonomic habits with low use of protective practices (Table 4). Inadequate ergonomic habits were common, with most students having poor viewing conditions.

### 3.5. Determinants of CVS Among Participants

Multivariable analysis found a number of important independent predictors of CVS. Students who were ≥24 years old had significantly higher odds of CVS (aOR = 9.73, 95% CI: 1.53–19.65, *p* = 0.046) than the reference group (18–20 years). Between academic courses, Arts and Humanities students had significantly reduced odds of CVS (aOR = 0.19, 95% CI: 0.05–0.64, *p* = 0.011) when compared to Applied Medical Sciences students.

Interestingly, a dose–response relationship was present for electronic device usage time. In comparison to pupils utilizing devices 1–2 h per day, increasingly higher chances of CVS were observed: 3–4 h (aOR = 4.13, 95% CI: 1.13–5.57, *p* = 0.032), 5–6 h (aOR = 5.31, 95% CI: 1.46–9.86, *p* = 0.011), and ≥7 h (aOR = 6.25, 95% CI: 1.74–8.01, *p* = 0.005). Sex had a non-significant trend, with males having lower chances of CVS than females. The ultimate model accounted for 23.4% of the variance in CVS occurrence and had a good statistical fit (Table 5).

## 4. Discussion

Epidemic-level CVS prevalence among university students is a focus of this research study, and it uncovers new risk patterns requiring urgent public health action. The prevalence rate of Computer Vision Syndrome among the participants was 89.7%. This is well above reported global averages of 66–69% from systematic reviews [18] and more recent meta-analyses reporting prevalence rates of 69.0% and prevalence rates highest among university students at 76.1% [19,20]. Three implications are notable: (1) a statistically significant dose–response trend of device use duration and CVS risk (p for trend = 0.002), (2) remarkable inter-disciplinary heterogeneity between 95.3% (Computer Sciences) and 75.4% (Arts and Humanities), and (3) extremely elevated risk in the >24-year-old students (OR = 9.73). These results are the highest CVS prevalence ever recorded in a Middle Eastern population of university students and are strong evidence for interventions targeting CVS.

The very high prevalence is consistent with earlier studies emphasizing the rising rate of CVS among students and professionals secondary to prolonged use of electronic devices, although our results surpass most published rates. A study investigating CVS prevalence among university students reported 64.36% [19], while the occurrence of digital eye strain has been reported to range from 5% to 65% in the pre-COVID-19 era, indicating our post-pandemic findings represent a substantial increase, likely reflecting intensified digital device dependency during and after remote learning periods [21]. Additionally, the prevalence of 89.7% is significantly higher than that described in earlier Saudi research among the general population as 77.6% [14] and college students as 82.2% [15], implying either regional differences or pandemic-driven acceleration in CVS incidence. Differences in prevalence across studies may also reflect methodological variation, including differences in case definitions, questionnaires used (e.g., CVS-Q vs. symptom checklists), and sample characteristics, in addition to pandemic-related changes in digital exposure.

### 4.1. Dose–Response Relationship and Causal Evidence

Statistically significant dose–response relationships provide strong evidence of a causative relationship between long-term device exposure and the development of CVS. The progressive risk is raised 4.13-fold for 3–4 h, 5.31-fold for 5–6 h, and 6.25-fold for ≥7 h versus 1–2 h per day. It shows evident biological plausibility and allows precise intervention targeting. It is the first study to define such an evident gradient effect in a university sample, with each additional category of use roughly doubling CVS risk. These results are consistent with earlier studies acknowledging extended computer exposure as a risk factor of significance, wherein computer users for greater than eight hours a day exhibited heightened CVS susceptibility [7]. The dose–response relationship accords with biological mechanisms of CVS, such as diminished blink rates, accommodative tension, and constant near-vision demands that increasingly become worse with longer duration [22].

The gradient relationship illustrates that device users for ≥7 h per day have CVS risk six or more times greater than minimal users, and that clinicians now have evidence-based cut points against which to compare patients’ device use. This result has direct clinical application, as 75% of our population had daily device use exceeding 5 h, and thus three-quarters of university students represent high-risk groups in need of targeted preventive interventions.

### 4.2. Academic Discipline Variations: Novel Risk Patterns

The 20-percentage point difference between academic fields is a newly identified risk pattern with important implications for targeted interventions. Students in Computer Sciences had the greatest prevalence at 95.3%, followed by Medical students at 94.8%, with Arts and Humanities students having the lowest prevalence at 75.4% with a protective association (OR = 0.19, *p* = 0.011). This result is consistent with earlier studies showing that Computer Science and Medical students shared 64.36% CVS prevalence, of which more than 20% had severe symptoms [23]. Computer Sciences, Business, and Medical students’ greater prevalence is due to extended screen use, heavy digital work, and heavy academic load involving constant device use for coding, financial analysis, and online medical materials [24].

By contrast, Arts and Humanities students probably enjoy more evenly distributed screen and non-screen academic activities, such as conventional reading, writing, and creative production, reducing overall CVS risk. Another study identified the effect of remote learning from COVID-19, reporting significant CVS symptom increases among university students, Business students being 1.6 times more susceptible than medical students to CVS [16]. These program-type variations imply that CVS development is substantially impacted by curriculum design and learning approaches in academic programs, making prevention efforts specifically targeting high-risk academic programs necessary.

### 4.3. Age-Related Risk Factors

Participants aged 24 years or above showed significantly increased odds of CVS development (OR = 9.73, *p* = 0.046), one of the strongest individual risk factors for CVS development in this study. This result surpasses earlier reports in which 26–35-year-old participants had 2.6 times greater odds than younger age groups [25]. The high risk among older students can be attributed to several physiological and behavioral processes: less tear secretion, diminished accommodative flexibility, longer visual strain from digital screens, from greater professional demands, and age-related changes in visual accommodation and convergence functions.

Earlier research has indicated that 67.4% of computer professionals with a mean age of 30.8 years suffered from CVS due to prolonged device use of more than 5 h, whereas individuals aged ≥25 years presented increased vulnerability due to alterations in physiology impacting tear film stability and accommodative capacity [24]. The approximately 10-fold higher risk in our study group indicates that even small increases in age above normal undergraduate ages considerably enhance CVS susceptibility, probably as a result of early presbyopic changes and escalating academic or professional pressures necessitating longer periods of screen use.

### 4.4. Symptom Profile and Clinical Associations

The presence of a strong correlation was found between headaches and use of the computer, with 68% of students experiencing headaches associated with electronic device use (*p* < 0.01). This result supports studies proving an 80% correlation of CVS with insomnia, and 53% with migraines from the use of electronic devices [21]. Electronic device headaches are mostly induced by prolonged exposure to blue light and screen glare, resulting in eye strain, dryness, and fatigue. Furthermore, repeated near-focus activity puts excessive demands on ciliary muscles, causing tension-type headache [26].

Past research has indicated that extended device use of over five hours a day increases the risk of CVS symptoms by far, such as headaches (66.5%) and dry eyes (51.5%), which predict greater risk in comparison to those with lower usage [21]. The symptom complex among our population indicates that CVS is a multisystem disorder whose impact is not limited to ocular comfort but also extends to neurological and musculoskeletal systems, necessitating holistic management strategies in place of sole ophthalmological treatments.

### 4.5. Protective Measures and Intervention Insights

Failure to use anti-glare screens was strongly related to CVS (*p* < 0.05), in line with earlier findings where monitor use without anti-glare treatment was a noteworthy risk factor (OR = 3.1) [27]. Anti-glare screens work by suppressing reflections and low-pass filtering high-energy visible light to prevent visual discomfort and possible retinal stress. Yet, they concur with recent evidence from a detailed Cochrane systematic review that blue-light blocking lenses do not reduce eye strain symptoms in the short term compared to non-filtering lenses significantly (*p* = 0.66) [28].

The American Academy of Ophthalmology has also discovered that blue light-blocking glasses have no substantial effect on digital eye strain symptoms, as present evidence indicates that though these lenses decrease glare by 68.8% and block 81.3% of blue light, they fail to impact significant contributing factors, including screen brightness, viewing distance, sustained focus, and improper workstation ergonomics [29]. These results indicate that environmental adaptations and behavioral treatments could be more effective than optical corrections for CVS prevention.

### 4.6. Public Health Implications and Clinical Applications

This study highlights that CVS is a growing concern among university students, demonstrating a clear dose-dependent relationship with screen time and discipline-specific effects, requiring urgent response by university health services and policymakers. The epidemic-level prevalence of about 9 in 10 students, along with evident dose–response relations, calls for urgent campus-level interventions. The analysis of population attributable risk implies that decreasing device use from ≥7 to <5 h per day would be able to prevent about 40% of CVS episodes in this group.

These findings provide critical insights for guiding targeted interventions. University health services must give the highest priority to CVS screening and prevention programs, with special focus on high-risk fields such as Computer Sciences and Medical courses. Enforcement of the 20-20-20 rule must be advocated through campaigns across the university, and ergonomic computer workstations with adequate lighting and monitor placement must be integrated into computer labs and study spaces. The dose–response relationship offers evidence-based targets for intervention design to allow clinicians to prescribe specific use advice in place of general advice.

### 4.7. Study Limitations and Future Directions

The strength of this study is the incorporation of several academic fields to enable an extensive comparison of CVS prevalence across various groups of students. The employment of standardized tools and statistical control to determine correlations ensures greater reliability of the findings. The large sample size (n = 427) with equal gender representation ensures sufficient power to ascertain significant associations. Despite these strengths, this study has several limitations that should be acknowledged. First, its cross-sectional design limits the establishment of causality or temporality between exposures and outcomes. Second, reliance on self-reported questionnaire data may have introduced recall or social desirability bias, which could affect the accuracy of symptom reporting. Third, the absence of objective ophthalmic assessments, such as refraction or ocular surface evaluations, limits the ability to validate participants’ reported symptoms. In addition, selection bias may exist since only six colleges were included, which might reduce the generalizability of the findings to the wider university population. Moreover, some potential confounders, including ambient lighting, workstation ergonomics, and lifestyle factors, were not comprehensively assessed and may have influenced the results. These limitations should be considered when generalizing the findings to all university students.

Longitudinal assessments in future research should be used to measure the long-term effects of electronic device usage on ocular health and academic achievement. Intervention studies that measure the efficacy of different preventive interventions in alleviating CVS symptoms among students would yield important evidence for educational practice. Examination of the association between the severity of CVS and academic achievement by discipline could guide education policy.

## 5. Conclusions

This study revealed a high prevalence of Computer Vision Syndrome (CVS) among university students, with clear dose–response patterns related to device-use duration and marked variation across academic disciplines. These findings underscore the urgent need for awareness programs, ergonomic education, and institutional policies promoting safer screen-use practices within academic settings. While the study’s cross-sectional design limits causal inference, its large and diverse sample provides robust evidence to guide future preventive strategies. Longitudinal research is warranted to confirm these associations and assess the long-term visual and academic impacts of extended digital exposure.

## Figures and Tables

**Table 1:** Demographic and Academic Characteristics of Study Participants (n = 427).

Characteristics	Frequency (%)	95% CI
**Age Groups**		
18–20 years	149 (34.9%)	30.4–39.4
21–23 years	251 (58.8%)	54.2–63.4
≥24 years	27 (6.3%)	4.1–8.5
**Sex**		
Female	215 (50.4%)	45.7–55.1
Male	212 (49.6%)	44.9–54.3
**Academic Discipline**		
Computer Sciences IT	86 (20.1%)	16.3–23.9
Business Administration	81 (19.0%)	15.3–22.7
Medicine	77 (18.0%)	14.4–21.6
Applied Medical Sciences	63 (14.8%)	11.4–18.2
Arts and Humanities	61 (14.3%)	10.9–17.7
Engineering	59 (13.8%)	10.5–17.1
**Academic Performance (GPA)**		
High (≥4.0)	295 (69.1%)	64.7–73.5
Moderate (3.0–3.99)	114 (26.7%)	22.5–30.9
Low (<3.0)	18 (4.2%)	2.4–6.0

**Table 2:** CVS Prevalence and Distribution by Academic Discipline (n = 427).

Academic Discipline	Total Students	CVS Cases	CVS Prevalence	95% CI	*p*-Value *
Computer Sciences IT	86	82	95.3%	89.1–99.5	
Medicine	77	73	94.8%	88.2–100.0	
Applied Medical Sciences	63	59	93.7%	86.8–100.0	
Business Administration	81	71	87.7%	79.8–95.6	<0.001
Engineering	59	52	88.1%	78.5–97.7	
Arts and Humanities	61	46	75.4%	64.1–86.7	
Overall	427	383	89.7%	86.8–92.6	

* Chi-square test for association between academic discipline and CVS.

**Table 3:** Dose–Response Relationship: Device Usage Duration and CVS Prevalence (n = 427).

Daily Usage Duration	Total Students	CVS Cases	CVS Prevalence	95% CI	Crude OR	95% CI	*p*-Value
1–2 h	25	16	64.0%	44.1–83.9	1.00	Reference	
3–4 h	82	72	87.8%	80.0–95.6	4.05	1.42–11.54	0.009
5–6 h	133	121	90.9%	85.7–96.1	5.68	2.12–15.24	0.001
≥7 h	187	173	92.5%	88.4–96.6	7.15	2.73–18.72	<0.001

**Table 4:** Electronic Device Usage Practices and Protective Behaviors (n = 427).

Usage Practices	Frequency (%)	95% CI	CVS Association (*p*-Value)
Device Positioning			
Below eye level	240 (56.2%)	51.5–60.9	0.847
At eye level	167 (39.1%)	34.5–43.7	
Above eye level	20 (4.7%)	2.7–6.7	
Viewing Distance			
<40 cm	169 (39.6%)	34.9–44.3	0.421
40 cm	193 (45.2%)	40.5–49.9	
>40 cm	65 (15.2%)	11.8–18.6	
Environmental Factors			
Bright environment usage	305 (71.4%)	67.0–75.8	0.879
Maximum brightness usage	97 (22.7%)	18.7–26.7	0.780
Protective Measures			
20-20-20 rule adherence	143 (33.5%)	29.0–38.0	0.569
Anti-glare screen usage	98 (23.0%)	18.9–27.1	0.013
Blue-light glasses usage	69 (16.2%)	12.7–19.7	0.810
Eye Care Practices			
Headaches during usage	273 (63.9%)	59.4–68.4	<0.001
Artificial tear drops usage	84 (19.7%)	15.9–23.5	0.142
Corrective eyewear usage	124 (29.0%)	24.7–33.3	0.025

**Table 5:** Multivariate Analysis: Independent Risk Factors for CVS (n = 427).

Risk Factors	Adjusted OR *	95% CI	*p*-Value
Age Groups (Reference: 18–20 years)			
21–23 years	1.47	0.68–3.14	0.321
≥24 years	9.73	1.53–19.65	0.046
Sex (Reference: Female)			
Male	0.49	0.22–1.08	0.079
Academic Discipline (Reference: Applied Medical Sciences)			
Arts and Humanities	0.19	0.05–0.64	0.011
Business Administration	0.39	0.09–1.38	0.161
Computer Sciences IT	2.11	0.43–10.83	0.352
Engineering	0.57	0.13–2.23	0.430
Medicine	0.83	0.17–3.87	0.803
Device Usage Duration (Reference: 1–2 h)			
3–4 h	4.13	1.13–5.57	0.032
5–6 h	5.31	1.46–9.86	0.011
≥7 h	6.25	1.74–8.01	0.005

* Wald X^2^ = 46.8, *p* < 0.001, Pseudo R^2^ = 0.234.

## Data Availability

The data supporting this study are not publicly available due to confidentiality and privacy considerations of the participants. However, the data may be made available by the corresponding author upon reasonable request.

## Supplementary Materials

The following supporting information can be downloaded at https://www.mdpi.com/article/10.3390/healthcare13212798/s1. File S1: Computer Vision Syndrome (CVS) Questionnaire.

## Abbreviations

The following abbreviations are used in this manuscript:

CVS	Computer Vision Syndrome
ED	Electronic Device
GPA	Grade Point Average
OR	Odds Ratio
CI	Confidence Interval
ICC	Intraclass Correlation Coefficient
STROBE	Strengthening the Reporting of Observational Studies in Epidemiology
DES	Digital Eye Strain
ADHD	Attention Deficit Hyperactivity Disorder
MSDs	Musculoskeletal Disorders
