# Supplementary material for: Computer Vision Syndrome Among Saudi University Students: A Cross-Sectional Analysis of Risks and Discipline Variations
Source: Healthcare (Basel). 2025 Nov 4;13(21):2798. doi: 10.3390/healthcare13212798 (PMC12609833; doi:10.3390/healthcare13212798)
Supplement: Supplementary file 1 [file healthcare-13-02798-s001.zip › healthcare-3940031-supplementary.pdf]

Computer Vision Syndrome (CVS) Questionnaire

This questionnaire is designed to assess digital device use, eye care practices, and symptoms of Computer Vision Syndrome among university students. Please answer all questions. Your responses will remain confidential.

Sociodemographic Information

1. Age ____ years

2. Gender ☐ Male ☐ Female

3. College ☐ Computer Sciences & IT ☐ Business Administration ☐ Medicine ☐ Applied Medical Sciences ☐ Arts & Humanities ☐ Engineering ☐ Other

4. GPA ☐ High (≥4.0) ☐ Moderate (3.0–3.99) ☐ Low (<3.0) ☐ Prefer not to say

Device Use

5. On a typical day, how many hours do you use electronic devices? ☐ 1–2 h ☐ 3–4 h ☐ 5–6 h ☐ ≥7 h

Ergonomics

6. Device position relative to eyes ☐ Below eye level ☐ At eye level ☐ Above eye level

7. Viewing distance ☐ <40 cm ☐ ≈40 cm ☐ >40 cm ☐ Not sure

Environment

8. Do you usually use devices in a bright environment? ☐ Yes ☐ No

9. Do you often keep the screen at maximum brightness? ☐ Yes ☐ No

Protective Behaviors

10. Do you follow the 20-20-20 rule? ☐ Yes, regularly ☐ Sometimes ☐ No

11. Do you use an anti-glare screen/filter? ☐ Yes ☐ No

12. Do you use blue-light blocking glasses? ☐ Yes ☐ No

Eye Care Practices

13. Do you experience headaches during or after device use? ☐ Yes ☐ No

14. Do you use artificial tear drops? ☐ No ☐ Yes, over-the-counter ☐ Yes, prescribed

15. Do you wear glasses or contact lenses? ☐ No ☐ Glasses ☐ Contact lenses ☐ Both

16. If yes, reason ☐ Myopia ☐ Hyperopia ☐ Astigmatism ☐ Other

CVS Symptoms (0–5 Likert scale)

17. Watery/teary eyes ☐0 ☐1 ☐2 ☐3 ☐4 ☐5

18. Dryness ☐0 ☐1 ☐2 ☐3 ☐4 ☐5

19. Irritation/burning ☐0 ☐1 ☐2 ☐3 ☐4 ☐5

20. Retro-bulbar pain (pain behind eyes) ☐0 ☐1 ☐2 ☐3 ☐4 ☐5

21. Eye strain/fatigue ☐0 ☐1 ☐2 ☐3 ☐4 ☐5

22. Redness ☐0 ☐1 ☐2 ☐3 ☐4 ☐5

23. Blurred vision ☐0 ☐1 ☐2 ☐3 ☐4 ☐5

24. Hot sensation in eyes ☐0 ☐1 ☐2 ☐3 ☐4 ☐5

25. Headache ☐0 ☐1 ☐2 ☐3 ☐4 ☐5
